# A patient with severe metformin-associated lactic acidosis complicated by acute coronary syndrome: a case report

**DOI:** 10.1186/s12882-022-02781-z

**Published:** 2022-05-06

**Authors:** N. Mammadova, J. Soukup, P. Shkodivskyi, C. Gudowski, A. Ahmed, R. U. Pliquett

**Affiliations:** 1 Department of Nephrology and Diabetology, Carl-Thiem Hospital, Cottbus, Germany; 2 Department of Anaesthesiology and Intensive Care, Carl-Thiem Hospital, Cottbus, Germany

**Keywords:** Metformin, Lactic acidosis, Acute coronary syndrome, Hemodialysis

## Abstract

**Introduction:**

Metformin-associated lactic acidosis (MALA) is a rare but life-threatening condition. Here, we report the outcome of a patient with MALA complicated by acute coronary syndrome.

**Case presentation:**

A 47-year-old obese woman of Caucasian ethnicity was admitted for syncope and tachypnea with Kussmaul breathing. She had a type-2 diabetes and was on oral antidiabetic therapy. Hemoglobin A1c was 6.6%. On admission, a severe acute kidney injury (serum creatinine: 1251 µmol/L) with hyperkalemia (7.5 mmol/L) and severe lactic acidosis (ph:7.042, bicarbonate: 9.9 mmol/L, partial pressure of carbon dioxide: 21.8 mmHg, lactate: 20.0 mmol/L) was found. Despite bicarbonate therapy, ph further decreased. Within 2.5 h of admission, a temporary hemodialysis catheter was placed, and one session of a high-efficiency hemodialysis was performed. 8 h after admission, a continuous venovenous hemodiafiltration was initiated and maintained for 2 days. The metformin therapy was stopped. Supplemental oxygen, intravenous catecholamines (4 days) and antibiotic therapy (7 days) were applied. During this therapy of lactic acidosis, an acute coronary syndrome evolved by day 2 after admission and resolved by day 5 in hospital. After recovery, the patient was transferred to a general ward on day 7 and left the hospital on day 11. By discharge, both the acute kidney injury and the acute coronary syndrome were reversible.

**Conclusion:**

In the patient with MALA complicated by acute coronary syndrome, the combination of a high-efficiency hemodialysis and, consecutively, continuous venovenous hemodiafiltration led to a favorable outcome.

## Background

Besides stopping metformin therapy, the use of sustained, low-efficiency dialysis (SLED) is associated with an improved outcome in patients with metformin-associated lactic acidosis (MALA), when compared to continuous venovenous hemodiafiltration (CVVHDF) technique as a standard therapy [1]. However, mortality rate of 21.4% is still exceedingly high [1]. Here, we report the outcome of a patient with severe MALA complicated by acute coronary syndrome having received intermittent hemodialysis and, consecutively, CVVHDF.

## Case presentation

A 47-year-old obese woman of Caucasian ethnicity (weight: 115 kg, height: 1.67 m, body-mass index: 41 kg/m^2^) presented in an emergency room for syncope and tachypnea with Kussmaul breathing pattern. The patient had a type-2 diabetes treated with oral antidiabetics, arterial hypertension, and chronic kidney disease (Kidney Disease: Improving Global Outcomes stage G3a). She fasted repeatedly for 7 days in a row to attempt weight loss. Following a fasting period one year earlier, exsiccosis leading to unconsciousness prompted an emergency-room visit without hospitalization. At the time of this visit, serum creatinine was 112 µmol/L (estimated glomerular filtration rate: 49.1 ml/min/1.73 m^2^).

**Fig. 1:**
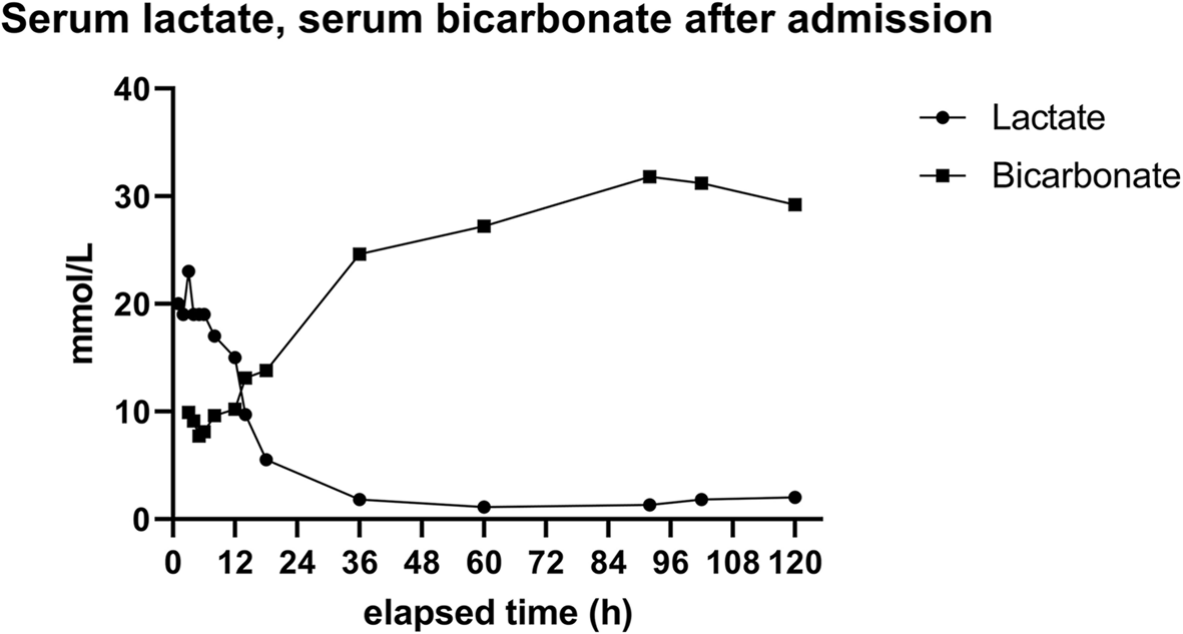
Serum lactate and serum bicarbonate measurements during evolving metformin-associated lactic acidosis since admission

At the current presentation, the patient felt unwell shortly after starting the fasting period and vomitted repeatedly. She, however, continued to take her medications including sitagliptin 50 mg BID, metformin 1 g BID, candesartan 16 mg BID, hydrochlorothiazide 12.5 mg QD and metoprolol 47.5 mg BID. A drug overdose was denied. In addition, the patient reported that diuresis declined as she was unable to drink sufficiently prior to hospitalization. On physical examination, the patient showed signs of exsiccosis, and moist rales over both lungs. The respiratory rate was 24 per minute, temperature was 36.7 °C. Laboratory results proved a severe acute kidney injury complicated by severe lactic acidosis being consistent with MALA (Table 1). In addition, a chest X-ray showed signs of pulmonary congestion, an evolving alveolar pulmonary edema and suspected pulmonary infiltrates in the right lung. Ultrasound of the kidneys revealed no abnormalities, especially no acute renal obstruction. There were no signs of venous congestion as the inferior caval vein was not dilated. For the treatment of MALA,, 100 ml sodium bicarbonate solution (8.4%) was applied 3 times without resolving the lactic acidosis, supplemental nasal oxygen (3 L/min) was provided, and the patient was transferred to the nephrology department for placment of a temporary dialysis catheter within 2.5 h of admission. Then, a high-efficiency hemodialysis using a single-use, high-flux polyethersulfone dialyzer (Revaclear 300, Baxter, IL, USA) with an effective surface of 1.4 m^2^ was initiated and maintained for 1 h. Plasma potassium was 5.5 mmol/L prior to hemodialysis, dialysate potassium concentration was 2 mmol/L. The counter-current flow rates of hemodialysate and blood were 500 mL/min and 250 mL/min, respectively. As for anticoagulation therapy, a bolus of 2000 units of unfractionated heparin was given intravenously. Blood pressure was 82/48 mmHg prior to hemodialysis, 71/41 mmHg afterwards. In order to avoid a rapid alleviation of uremia, the intermittent hemodialysis was paused after 1 h of treatment, and the patient was transferred to an intermediate care unit. There, 8 h post admission, a CVVHDF using a single-use acrylonitrile-copolymer dialyzer (M150, Baxter, IL, USA) with an effective surface of 1.5 m^2^ was started and maintained for 46 h. Hemodialysate flow rate was 1.2 L/h or 20 mL/min; the respective effluent rate was 10.4 mL/kg/h. Potassium in the hemodialysate was 4 mmol/L. An ultrafiltration rate of 0 to 50 ml/hour was applied initially (1 L ultrafiltration on day 1). After 24 h, ultrafiltration was stopped because the patient complained of muscle cramps. Intravenous antibiotics including piperacillin (4 g/) and tazobactam (0.5 g) thrice daily were administered for treatment of suspected sepsis. The capillary-blood lactate and bicarbonate concentrations stabilized during the initial 24 h (Fig. 1). However, by day 3 in hospital, serum sodium still increased to a maximum of 149 mmol/L. Although the patient was allowed to drink ad libitum*,* intravenous rehydration had to be applied to compensate for the polyuria. In addition norepinephrine was infused (0.01 – 0.05 µg/kg body weight/h) from day 2 to day 4 for arterial hypotension. Respiratory rate peaked at 34/min during the first 4 days in hospital. As the oxygen saturation remained stable, supplemental oxygen was discontinued on day 5.

On day 2 in hospital, during ongoing renal-replacement therapy, the patient experienced an acute coronary syndrome with a rise of troponin T from 57.6 ng/L on day 1 to 632 ng/L on day 2 in hospital. Troponin T peaked off from day 4 on (172 ng/L on day 4, 158 ng/L on day 5). Creatinine kinase did not rise at any time. N-terminal pro-brain natriuretic peptide (NT pro-BNP) was determined on day 2 in hospital for the first time (1095 pmol/L), rose to 3043 pmol/L on day 3 and fell off by day 4 (2314 pmol/L) and day 5 (757 pmol/L). Electrocardiogram showed a ST-depression by 0.1 mV in leads II, III, aVF, transthoracic echocardiography, performed on the day of discharge, proved a normal left-ventricular function without regional hypokinesia. Laboratory testing for vasculitis was unrevealing. Therapeutic anticoagulation with unfractionated heparin was maintained for 6 days. Blood pressure remained stable, and, after stopping renal-replacement therapy, the acute kidney injury reversed. Blood and urine cultures taken prior to antibiotic therapy remained negative. Laboratory results (C-reactive protein, procalcitonin, leukocyte count) were not consistent with an infection or sepsis as a possible cause of lactic acidosis. As for medication, oral antidiabetics were stopped upon admission. Sitagliptin (25 mg/d) was reintroduced on day 3. The dose was increased to 50 mg/d at the time of discharge.

The patient fully recovered and left the hospital on the 11^th^ day following admission. At discharge, patient weight was 108.3 kg, body-mass index 38.8 kg/m^2^. In a follow-up call with the patient 18 months later, the patient had no complaints and reported no rehospitalization since discharge. Sitagliptin (50 mg/d) was maintained as oral diabetes medication.

## Discussion and conclusions

Here, the combination of prolonged fasting and vomiting likely caused the severe acute kidney injury leading to the occurrence of severe MALA. Despite a preexisting moderate chronic kidney disease, the metformin dose was not adjusted prior to index hospitalization nor was it withheld during the fasting period. An initial high-efficiency hemodialysis over 1 h was chosen to effectively treat lactic acidosis because intravenously applied 8.4% sodium-bicarbonate infusions (3 × 100 mL) had no effect on serum levels of bicarbonate, while serum lactate continued to rise to a maximum of 23 mmol/L. In comparison to CVVHDF, the dialysate flow rate was 25 times higher in intermittent hemodialysis (20 mL/min versus 500 mL/min). In theory, to reach the goal of a minimum effluent rate of 35 mL/kg/h, the hemodialysate-flow rate in CVVHDF would have to exceed 4 L/h in this patient.

In lactic acidosis, recent evidence has attributed more advantages to the SLED in comparison with CVVHDF [1]. The use of a short-term intermittent hemodialysis as performed here allowed for an even more effective systemic clearance of metformin. Specifically, SLED allows for a maximal hemodialysate-flow rate of 250 mL/min, which is 12.5 times higher than that of CVVHDF in our case, however, half of the hemodialysate-flow rate in intermittent hemodialysis (500 ml/L). The initial intermittent hemodialysis followed by CVVHDF, therefore, normalized the severe MALA within the first 24 h of admission.

However, based on increasing troponin T levels, the patient experienced an acute coronary syndrome with specific ECG changes that were consistent with a posterior-wall ischemia. The long-term effects of diabetes may explain the silent myocardial ischemia in this case. The proven NT pro-BNP elevation supports the diagnosis of an acute coronary syndrome [2]. Echocardiography could not prove any sequelae that persisted after the acute coronary syndrome. However, as a limitation, a coronary angiography was not performed in this patient. Therefore, a possible acute coronary thrombosis or plaque rupture could not be excluded or proven. As an alternative explanation, a severe metabolic acidosis represents one cause for an acute coronary syndrome by virtue of a decline in energy utilization [3]. Here, as an indirect evidence, the patient was discharged without any symptoms, once the acidosis had resolved. Alternatively, a coronary pathology involving a temporary coronary thrombosis is possible. In the literature, one patient was reported to have died of cardiac ischemia during MALA [4]. Another patient presented with ST-elevation myocardial infarction that resolved after treatment of MALA [5]. In these published cases and our present case, a direct effect of acidosis on cardiac metabolism is likely. In other MALA cases [6], hyperkalemia due to acute kidney injury may have aggravated the outcome. Apart from the acute coronary syndrome, the timely normalization of acidosis by a high-efficiency hemodialysis followed by CVVHDF appears to be the key for the good outcome in the present case. Specifically, the normalization of lactic acidosis coincided with an alleviation of acute coronary syndrome. As shown here and in previous cases [7, 8], both the cessation of metformin and supportive therapy including intravenously applied sodium bicarbonate did not suffice to control a severe MALA. Specifically, intensive-care-unit patients with severe MALA, in whom hemodialysis or CVVHDF was performed, had a similar mortality rate when compared to patients with less severe MALA not being treated by renal-replacement therapy [8]. This result implies a benefit in favor for renal replacement therapy in MALA. Likewise, a prolonged high-efficiency hemodialysis was sufficient to correct MALA [9]. However, in the present case, a prolonged hemodialysis was not feasible due to the risk of a dialysis disequilibrium syndrome. Rather, as shown here and in a previous case [10], a high-efficiency hemodialysis added an effective and quicker detoxification capacity to the standard of continuous renal replacement therapy allowing for a slow recovery from uremia, yet a swift normalization of lactic acidosis.

In summary, this case is consistent with the view that severe MALA triggered an acute coronary syndrome. During high-efficiency hemodialysis followed by CVVHDF, both MALA and troponin-T elevation normalized rapidly.

**Table 1:** Laboratory test results from hospital obtained on admission and at discharge

**Laboratory parameters**	**Unit**	**Reference** **range**	**Day 1 (before** **hemodialysis)**	**Day 1 (after** **hemodialysis)**	**Day of discharge**
Serum Sodium	mmol/L	136-145	140	145	141
Potassium	mmol/L	3.4-4.5	7.5	5.4	3.9
Glucose	mmol/L	4.11-6.05	5.7	8.8	6.4
eGFR	ml/min/1.73m²	> 60.00	N/A	N/A	41.1
Creatinine	µmol/L	62-106	1251	530	132
Urea	mmol/L	2.76-8.07	N/A	13.2	5.0
ALAT	µmol/l*s	< 0.6	0.22	N/A	N/A
ASAT	µmol/l*s	< 0.6	0.73	N/A	N/A
GGTP	µkat/L	< 5.0	N/A	N/A	N/A
Troponin T	ng/L	< 5.0	57.6	N/A	N/A
C-reactive protein	mg/L	< 1.00	6.4	N/A	3.5
Plasma Hemoglobin	mmol/L	7.5-9.6	8.2	8.0	5.6
Leukocyte count	Gpt/L	3.70-9.90	19.7	N/A	5.7
Platelet count	Gpt/L	140-360	512	N/A	417
D dimers	mg/L	<0.5	2.9	3.7	NA
International normalized ratio Capillary blood ph Oxygen saturation Bicarbonate Lactate	mmol/L mmol/L	0.85-1.15 7.37-7.45 94-98 21-26 1.1-1.9	1.54 7.042 81 9.9 23.0	1.18 7.075 76 9.6 17.0	NA NA NA NA NA

## Data Availability

The authors presented relevant information and data in the manuscript. A copy of all de-identified source data (case files in hospital) can be obtained upon reasonable request by the corresponding author. For legal reasons, the publication of all source data, even in a de-identified format, is not possible.

## Abbreviations

CVVHDF: Continuous venovenous hemodiafiltration
MALA: Metformin-associated lactic acidosis
NT pro-BNP: N-terminal pro-brain natriuretic peptide
SLED: Sustained, low-efficiency dialysis
